# An RNA-Seq-Based Framework for Characterizing Canine Prostate Cancer and Prioritizing Clinically Relevant Biomarker Candidate Genes

**DOI:** 10.3390/ijms222111481

**Published:** 2021-10-25

**Authors:** Heike Thiemeyer, Leila Taher, Jan Torben Schille, Eva-Maria Packeiser, Lisa K. Harder, Marion Hewicker-Trautwein, Bertram Brenig, Ekkehard Schütz, Julia Beck, Ingo Nolte, Hugo Murua Escobar

**Affiliations:** 1 Small Animal Clinic, University of Veterinary Medicine Hannover, Foundation, 30559 Hannover, Germany; heike.thiemeyer@icloud.com (H.T.); jan.torben.schille@tiho-hannover.de (J.T.S.); eva-maria.packeiser@tiho-hannover.de (E.-M.P.); lisa.harder@tiho-hannover.de (L.K.H.); ingo.nolte@tiho-hannover.de (I.N.); 2 Department of Hematology/Oncology/Palliative Care, Rostock University Medical Centre, 18057 Rostock, Germany; 3 Institute of Biomedical Informatics, Graz University of Technology, 8010 Graz, Austria; leila.taher@tugraz.at; 4 Institute of Pathology, University of Veterinary Medicine Hannover, Foundation, 30559 Hannover, Germany; marion.hewicker-trautwein@tiho-hannover.de; 5 Institute of Veterinary Medicine, University of Göttingen, 37077 Göttingen, Germany; bbrenig@gwdg.de; 6 Chronix Biomedical GmbH, 37079 Göttingen, Germany; esc@chronixbiomedical.de (E.S.); jbeck@chronixbiomedical.de (J.B.); 7 Comprehensive Cancer Center Mecklenburg-Vorpommern (CCC-MV), Campus Rostock, University of Rostock, 18057 Rostock, Germany

**Keywords:** canine prostate cancer, RNA-Sequencing, whole transcriptome analysis, candidate biomarker genes, animal model, molecular diagnostics

## Abstract

Prostate cancer (PCa) in dogs is a highly malignant disease akin to its human counterpart. In contrast to the situation in humans, multi-gene approaches facilitating risk stratification of canine PCa are barely established. The aims of this study were the characterization of the transcriptional landscape of canine PCa and the identification of diagnostic, prognostic and/or therapeutic biomarkers through a multi-step screening approach. RNA-Sequencing of ten malignant tissues and fine-needle aspirations (FNA), and 14 nonmalignant tissues and FNAs was performed to find differentially expressed genes (DEGs) and deregulated pathways. The 4098 observed DEGs were involved in 49 pathways. These 49 pathways could be grouped into five superpathways summarizing the hallmarks of canine PCa: (i) inflammatory response and cytokines; (ii) regulation of the immune system and cell death; (iii) cell surface and PI3K signaling; (iv) cell cycle; and (v) phagosome and autophagy. Among the highly deregulated, moderately to strongly expressed DEGs that were members of one or more superpathways, 169 DEGs were listed in relevant databases and/or the literature and included members of the PCa pathway, oncogenes, prostate-specific genes, and druggable genes. These genes are novel and promising candidate diagnostic, prognostic and/or therapeutic canine PCa biomarkers.

## 1. Introduction

Prostate cancer (PCa) in dogs can occur spontaneously, with aging dogs exhibiting higher incidence thereof than any other nonhuman species [1,2,3]. Although the incidence in dogs (0.2%, [4]) is substantially lower than in men, canine PCa is typically aggressive, with a high likelihood of metastasis [5]. In particular, the metastatic castration-resistant state of human PCa shares many clinical properties with canine PCa [5,6,7], making canine PCa of considerable value for the study of human cancer pathogenesis and for the evaluation of therapeutic interventions [8].

Despite some advances, there are gaps in our knowledge of canine PCa and its diagnostic workup [2,9], especially compared to human PCa. Notwithstanding numerous immunohistochemical and a few gene expression studies on canine PCa [10,11,12,13,14,15,16,17,18,19], no comprehensive marker set has proved suitable for routine clinical assessment [2,20]. With regard to the prostate-specific antigen (PSA, encoded by Kallikrein Related Peptidase 3 or *KLK3*), as commonly used blood serum biomarker in regular checkups for human PCa [21], no unambiguous canine ortholog has been identified for the human gene encoding PSA, i.e., *KLK3* [22]. The canine prostate-specific arginine esterase (CPSE, encoded by *KLK2*), another member of the kallikrein family with striking similarities to human PSA [23], has been suggested as a blood serum marker for diagnosing benign prostatic hyperplasia [24]. Whether CPSE is also a marker for diagnosing canine PCa still has to be investigated [25,26]. Further studies are needed to establish a reliable classification system and molecular diagnostic tests for canine PCa [9]. Histopathological terminology standards for prostatic diseases in dogs and also the Gleason score grading system used for human PCa, have been recently adapted to canine PCa [27]. Nonetheless, most canine PCa continues to be diagnosed at an advanced stage with limited therapeutic options [5,7].

Molecular information, especially that enabled by high-throughput technologies such as next-generation sequencing (NGS), plays an increasingly important role in the diagnostic work-up of human PCa [28,29]. In veterinary medicine, high-throughput technologies are emerging as the method of choice to characterize diseases at molecular level [30]. Recently, genome-wide profiling of androgen receptor (AR) negative canine PCa revealed a large number of copy number alterations associated with aberrant expression of cancer-related and tumor suppressor genes [31]. Nevertheless, most gene expression studies in canine PCa to date are based on the quantitative polymerase chain reaction (qPCR). They have provided information on the hormonal network of canine PCa, including the *AR*, or tumor suppressor genes, such as NK3 homeobox 1 (*NKX3-1*), and therapeutically relevant genes, like KIT proto-oncogene, receptor tyrosine kinase (*KIT*) [11,16,19]. However, although qPCR-based quantification is highly specific and sensitive, it is limited by its throughput and impracticable at whole-genome level [32]. In contrast to qPCR-based approaches, NGS technologies such as RNA-Sequencing (RNA-Seq) yield an almost fully comprehensive picture of the transcriptional landscape and have the potential to provide insight into the molecular mechanisms and regulatory networks underlying PCa [33,34,35].

Integrating the clinical findings with the molecular context is imperative in order to further characterize any disease [30,32,36] and prospectively enable the intelligent selection of interventional compounds. Thus, the combination of clinical, pathological and gene expression data has led to the development of various tissue-based multi-gene assays for human PCa [36]. Some such assays even enable the use of formalin-fixed paraffin-embedded needle core biopsies for gene expression profiling [36]. In dogs, we were recently able to show that fresh frozen tissue and aspirates collected *intra vitam* by fine-needle aspiration biopsies (FNA) of the canine prostate can serve as reliable sampling material for gene expression profiling in clinical settings [37]. Taking a step forward towards developing new molecular diagnostic approaches for canine PCa, the present study used RNA-Seq data of malignant and nonmalignant *post mortem* prostate tissues and *intra vitam* fine-needle aspirates to characterize the transcriptional landscape of canine PCa. A multidisciplinary approach was applied to identify canine PCa biomarker candidates that could enable targeting specific clinical questions and warrant further research. Ultimately, this study aims at improving the diagnostic and therapeutic possibilities for canine PCa and at strengthening the dog as a model for human PCa.

## 2. Results

### 2.1. Discriminating Malignant and Nonmalignant Canine Prostate Samples

**Figure 1:**
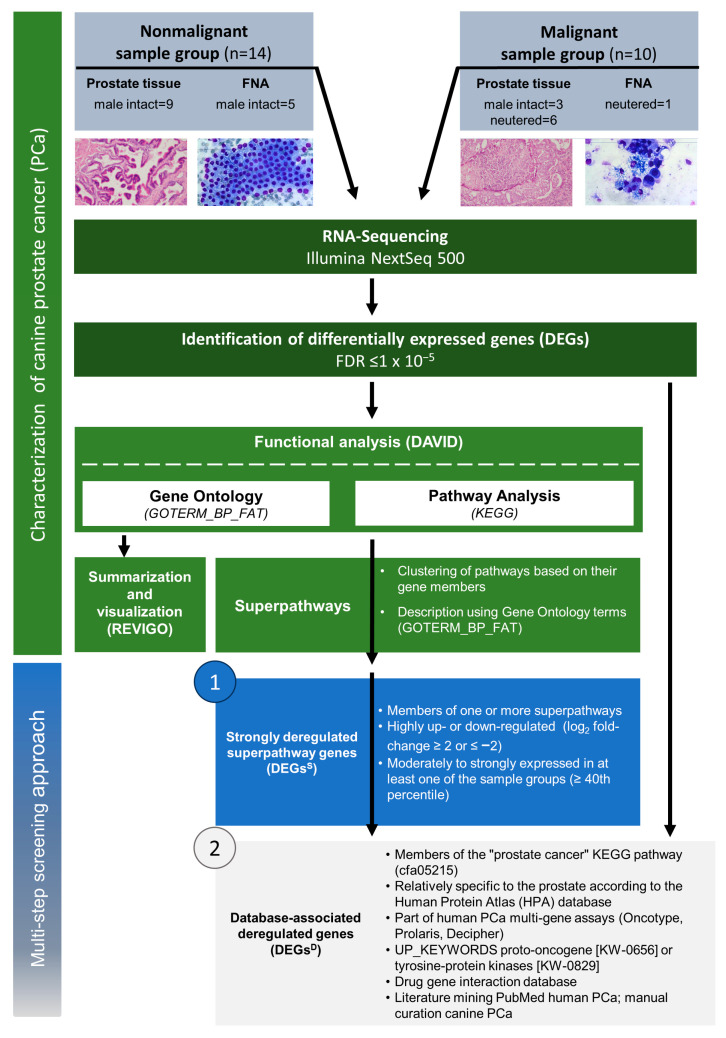
Experimental design. Flowchart of RNA-Sequencing (RNA-Seq) analysis for characterization (green) and identification of differentially expressed genes (DEGs) between malignant and nonmalignant canine prostate samples. Selection (blue and gray) of strongly deregulated superpathway genes (DEGs^S^) and database-associated deregulated genes (DEGs^D^).

**Figure 2:**
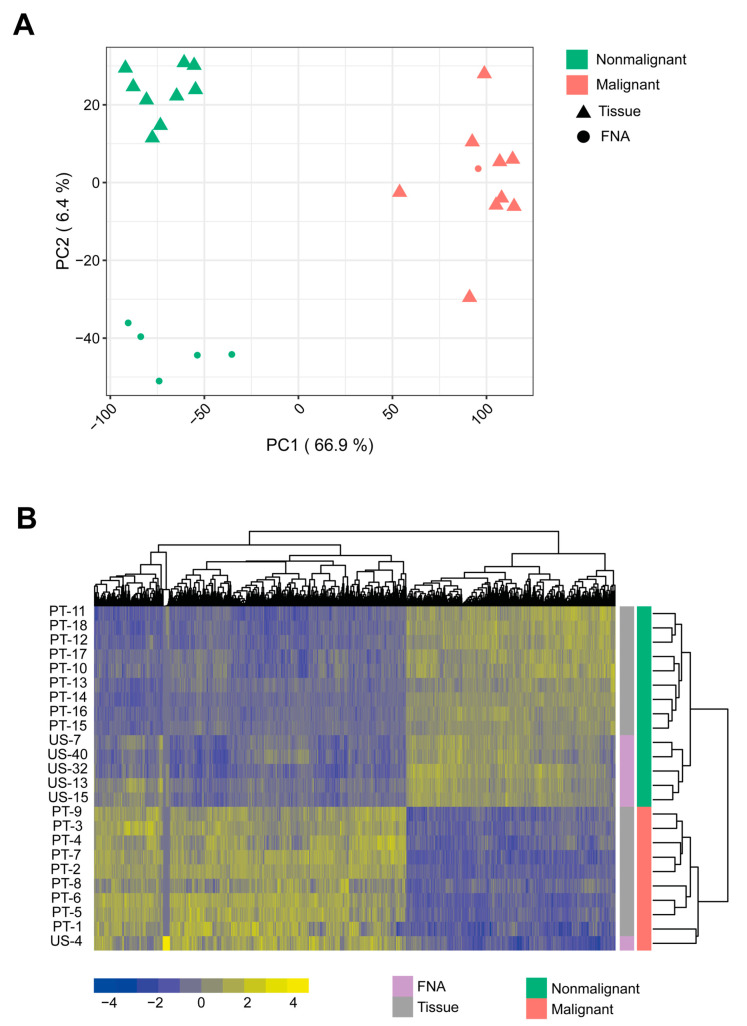
Differentially expressed genes (DEGs) between malignant and nonmalignant canine prostate samples. (**A**) Principal component analysis (PCA) based on the normalized regularized logarithm (rlog)-transformed read counts of the DEGs: fine-needle aspiration (FNA) samples (circle), prostate tissue samples (triangle), nonmalignant (green), malignant samples (red). PCA verified that most of the variance (66.9%, PC1) was associated with the altered expression between the malignant and the nonmalignant canine PCa samples; (**B**) Heatmap and hierarchical clustering of prostate tissue (gray), FNA (purple), nonmalignant (green) and malignant (red) samples and DEGs based on Euclidean distances between normalized rlog-transformed counts. Rows have been centered and scaled to compute z-scores.

RNA-Seq data of 10 malignant and 14 nonmalignant canine prostate tissue and FNA samples were compared to characterize the transcriptional landscape of the malignant phenotype of the canine prostate and identify canine PCa biomarker candidates featuring its most striking molecular mechanisms (Figure 1). Differential expression analysis revealed a total of 4098 DEGs between these two sample groups (see Materials and Methods). The transcriptomic profiles discriminated between malignant and nonmalignant samples according to the corresponding histopathological or cytological diagnoses (Figure 2). In particular, the differences between the two sample groups explained at least 66.9% of the variance in the data (Figure 2A). Most DEGs (2454, 60%) were upregulated. Specifically, 1665 DEGs (41%) were highly upregulated (log_2_ fold-change ≥ 2), while 839 DEGs (21%) were highly downregulated (log_2_ fold-change ≤ −2). Overall, 3328 DEGs were annotated with one or more biological processes (Figure S1A), mainly associated with immune response, cell activation and regulation of cell proliferation (Figure S1B, see Materials and Methods).

### 2.2. The Transcriptional Landscape of Canine PCa Is Characterized by the Deregulation of Pathways Involved in Immune Response, Cell Adhesion, PI3K Signaling, Cell Cycle, as Well as Phagosome and Autophagy

**Figure 3:**
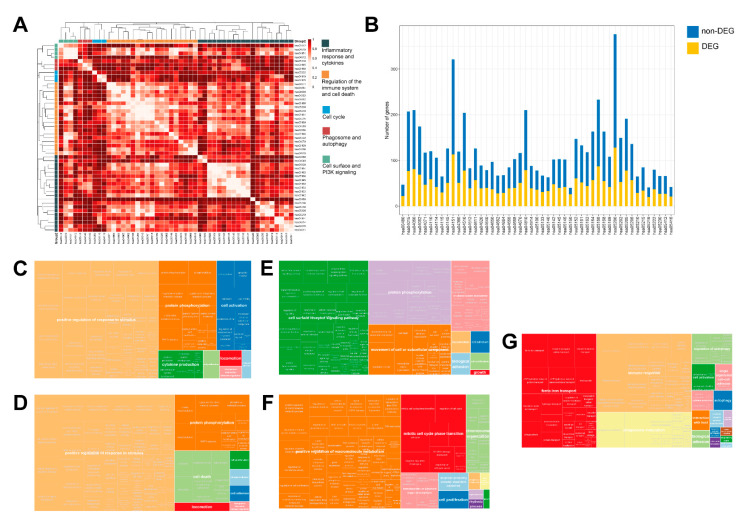
Superpathways summarizing the hallmarks of canine prostate cancer (PCa). (**A**) The forty-nine Kyoto Encyclopedia of Genes and Genomes (KEGG) pathways enriched among canine PCa differentially expressed genes (DEGs). Note that the analysis was performed based on the human homologs of the canine DEGs to take advantage of the better annotation available for human genes. The color of the cells in the heatmap visualizes the overlap coefficient-based distance between pairs of pathways; while 0 implies a 100% overlap between the gene members of the pathways, 1 implies 0%. The pathways were hierarchically clustered using the complete linkage algorithm based on a distance defined as 1 minus the overlap coefficient; (**B**) Stacked bar chart showing the number of gene members that are not differentially expressed (yellow) and DEGs (blue) in each of the 49 pathways enriched among canine PCa DEGs; (**C**–**G**) Superpathway treemaps: Summary of the Gene Ontology (GO) biological processes enriched in the five superpathways. Gene ontology (GOTERM_BP_FAT) enrichment analysis was performed on DEGs using the database for annotation, visualization and integrated Discovery (DAVID) [38], and clustered and displayed with Reduce + Visualize Gene ontology (REVIGO) [39]. The treemaps show the enriched biological processes and the box sizes represent the respective negative logarithm of the false discovery rate (FDR): (**C**) Inflammatory response and cytokines superpathway; (**D**) Regulation of the immune system and cell death superpathway; (**E**) Cell surface and PI3K signaling superpathway; (**F**) Cell cycle superpathway; (**G**) Phagosome and autophagy superpathway.

Pathway analysis of the 4098 DEGs revealed 49 enriched pathways (see Materials and Methods). Based on their cross-talk, which was quantified based on the overlap coefficient between the pathway gene members, the pathways could be grouped into five superpathways summarizing the disease hallmarks of canine PCa (Figure 3). Overall, the superpathways involved 1973 genes, with the two largest ones encompassing 85% of all genes.

All superpathways could be mapped to key biological processes commonly deregulated in cancer. The largest superpathway was associated with the biological processes “positive regulation of response to stimulus”, “protein phosphorylation”, “cell activation” and “cytokine production”, among others (see Materials and Methods, Figure 3C). It involved 20 pathways and 1182 genes. The second largest superpathway was related to “positive regulation of response to stimulus”, “protein phosphorylation” and “cell death” (Figure 3D). It comprised 19 pathways and 1075 genes. The third superpathway was connected to “cell surface receptor signaling pathway”, “protein phosphorylation”, “movement of cell or subcellular component” and “circulatory system development” (Figure 3E). This superpathway consisted of four pathways and 459 genes. The fourth superpathway was involved in “positive regulation of macromolecule metabolism”, “mitotic cell cycle phase transition”, “chromosome organization” and “hematopoietic or lymphoid organ development” (Figure 3F). It comprised three pathways and 289 genes. The smallest superpathway was linked to “ferric iron transport”, “immune response”, “phagosome maturation” and “regulation of autophagy” (Figure 3G). It included three pathways and 174 genes. Hereinafter, we refer to these five superpathways as (i) “inflammatory response and cytokines”, (ii) “regulation of the immune system and cell death”, (iii) “cell surface and PI3K signaling”, (iv) “cell cycle” and (v) “phagosome and autophagy”, respectively.

The mean of the median overlap coefficient across all pathways in a superpathway ranged from 4% (“cell cycle”) to 23% (“regulation of the immune system and cell death”), reflecting various levels of cross-talking between the pathways. Between 34% (“inflammatory response and cytokines”) and 43% (“phagosome and autophagy”) of the genes in each superpathway were differentially expressed. From a total of 688 DEGs in any of the five superpathways, the vast majority of DEGs (562, 82%) were upregulated. Moreover, all the pathways involved in the superpathways were upregulated in the sense that they comprised a larger number of upregulated genes than downregulated ones.

In particular, the “regulation of the immune system and cell death” superpathway included the “prostate cancer” pathway. This pathway has 84 gene members, of which 34 (40%) were DEGs. Interestingly, many of those DEGs are also members of three of the remaining four superpathways: 29 of the “inflammatory response and cytokines” superpathway, 27 of the “cell surface and PI3K signaling” superpathway and eight of the “cell cycle” superpathway, confirming its central role in PCa.

### 2.3. A Framework for Selecting Canine PCa Biomarker Candidates with Clinical Value

**Figure 4:**
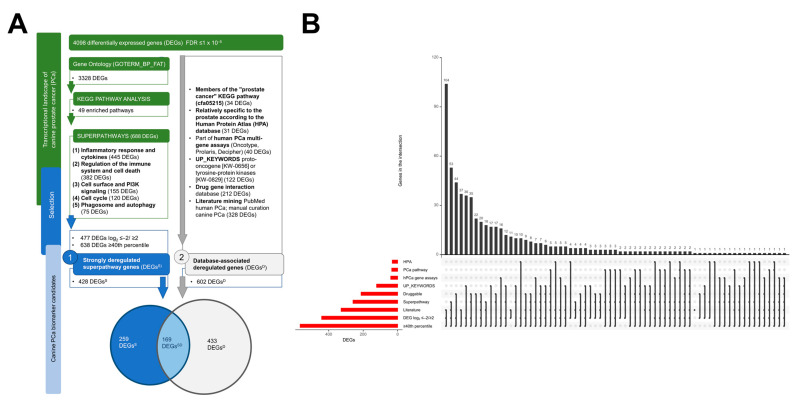
Intersections and selection of canine prostate cancer (PCa) biomarker candidates. (**A**) Flowchart indicating how selection of canine PCa biomarker candidates is performed based on two different approaches: (1) strongly deregulated superpathway genes (DEGs^S^) and (2) database-associated deregulated genes (DEGs^D^), with overlap DEGs^SD^. FNA: Fine-needle aspiration sample; KEGG: Kyoto Encyclopedia of Genes and Genomes; REVIGO: Reduce + Visualize Gene ontology; (**B**) V UpSet plot visualizing the intersection between different groups of differentially expressed genes (DEGs) according to the two different approaches. Criteria for DEGs^D^: the human protein atlas (HPA) database, the “prostate cancer” pathway of the Kyoto encyclopedia of genes and genomes (PCa pathway), human prostate cancer multi-gene assays (hPCa gene assays), UniProt keywords proto-oncogene [KW-0656] and tyrosine-protein kinases [KW-0829] (UP_KEYWORDS), druggable database and literature. Criteria for DEGs^S^: superpathway, strongly DEGs with base-2 logarithm (log_2_) fold-change ≤−2/≥2 and ≥40th percentile. Intersections of DEG^S^ and DEGs^D^ as selection criteria for DEGs^SD^. The horizontal bars (left, red) display the total number of DEGs in each group. The vertical bars (top, gray) visualize the number of DEGs in each combination of groups, as indicated by the dots. Note that all the intersections are disjoint.

Among the 688 DEGs making up the five superpathways, 477 were highly deregulated (see Materials and Methods, Figure 4A), with the vast majority of them (84%) being upregulated. Approximately 90% (428) of the highly deregulated genes were moderately to strongly expressed in one or both sample groups (with median library size-normalized counts above the 40th percentile; see Materials and Methods). These 428 genes are hereinafter referred to as DEGs^S^ (Figure 4A), and represent between 88% and 92% of the highly deregulated genes in each superpathway. Specifically, 65% (276) were members of the “inflammatory response and cytokines” superpathway, 54% (230) of the “regulation of the immune system and cell death” superpathway, 22% (92) of the “cell surface and PI3K signaling” superpathway, 16% (70) of the “cell cycle” superpathway and 12% (52) of the “phagosome and autophagy” superpathway.

Furthermore, of the 4098 DEGs, 602 (15%) were identified in relevant public databases or in the literature, and are hereinafter referred to as DEGs^D^ (Figure 4A). Among them, 328 DEGs were mentioned in the literature in the context of canine or human PCa, 212 were potentially modulated by small molecules (“druggable”), 122 DEGs were proto-oncogenes or tyrosine-protein kinases, 40 DEGs were in human PCa gene assays, 34 were members of the “prostate cancer” pathway, and 31 were relatively specific to the prostate according to the Human Protein Atlas (HPA) database (see Materials and Methods, Figure 4B). Only 20% (123) of the DEGs^D^ were found in two or more of these databases and no DEG^D^ was identified in any of them (Figure 4B). Furthermore, with the exception of the DEGs^D^ in the “prostate cancer” pathway, most DEGs^D^ in a given database were only present in that database. In contrast, the vast majority (76%, 26) of the differentially expressed members of the “prostate cancer” pathway were found in at least another database. Specifically, 25 of the DEGs^D^ in the “prostate cancer” pathway are “druggable” or have been reported in the literature in the context of canine or human PCa. Interestingly, only a few of the assays- (35%, 14) or literature-associated (30%, 100) DEGs^D^ could be linked to other databases.

Among the 602 DEGs^D^, 169 (28%) were also categorized as strongly deregulated superpathway genes (DEGs^S^) (Figure 4, Table S1). These 169 genes are hereinafter referred to as DEGs^SD^ (Figure 4A). Most (397) of the remaining 433 DEGs^D^ were moderately to strongly expressed in one or both sample groups and many (272) were highly deregulated; 90 were members of a superpathway (Figure 4B, Table S1). Only one DEG^D^ (pre-rRNA-processing protein TSR1 homolog) did not satisfy any of the criteria used for defining the DEGs^S^. Among the 169 DEGs^SD^, 19 were proto-oncogenes (*AURKA*, *CSF1R*, *EGFR*, *ETS1*, *ETV1*, *FGR*, *FOS*, *HCK*, *KIT*, *LYN*, *MECOM*, *MET*, *NRAS*, *PDGFRA*, *PDGFRB*, *PTTG1*, *REL*, *SPI1*, and *ZBTB16)*, nine were relatively specific to the (human) prostate (*ARG2*, *CHRM1*, *CREB3L4*, *CXCL10*, *KLK2*, *NKX3-1*, *SLC45A3*, *SRD5A2* and *TMPRSS2*), thirteen were orthologs to genes on commercial human PCa multi-gene assays (*BUB1B*, *CDK1*, *COL1A1*, *FOS*, *IQGAP3*, *KLK2*, *ORC6*, *PLK1*, *PTTG1*, *RAD51*, *RRM2*, *SRD5A2 and THBS2*), and 80 were druggable, including 75 (94%) upregulated genes (Figure 4B, Table S1). The orthologs of the genes on commercial human PCa multi-gene assays are associated with cell cycle progression (*CDK1*, *BUB1B*, *ORC6*, *RAD51*, *PLK1*, *PTTG1* and *RRM2*), cell proliferation (*IQGAP3*), stromal response (*COL1A1*), cell adhesion (*THBS2*), stress response (*FOS*), and androgen signaling (*KLK2*, *SRD5A2*). Of the 75 upregulated druggable DEGs^SD^, 15 (*BTK*, *CSF1R*, *EGFR*, *EIF2AK2*, *EPHA2*, *FGR*, *HCK*, *JAK3*, *KIT*, *LYN*, *MET*, *PDGFRA*, *PDGFRB*, *SYK* and *TEC*) encode tyrosine kinases (Table S1). Fifty-eight percent of the 169 DEGs^SD^ have been mentioned in the literature, with 45 DEGs^SD^ being present in at least one additional database (Figure 4B, Table S1). Specifically, half of the “prostate cancer” pathway DEGs^SD^ had previously been referred to in the literature (*AR*, *CDKN1A*, *CTNNB1*, *EGFR*, *KLK2*, *NKX3-1*, *PDGFRA*, *SRD5A2*) (Figure 4B, Table S1). The “prostate cancer” pathway also comprises genes that are relatively specific to the (human) prostate (*CREB3L4*, *KLK2*, *NKX3-1* and *SRD5A2*), proto-oncogenes (*EGFR*, *NRAS*, *PDGFRA* and *PDGFRB*), and druggable candidates (*CDKN1A*, *CTNNB1*, *EGFR*, *NRAS*, *PDGFRA*, *PDGFRB*, *PIK3CD* and *PIK3CG*) (Figure 4B, Table S1).

### 2.4. The Gene Network of 602 DEGs^D^ Form a Tightly Interconnected Gene Network Intertwined with the “Prostate Cancer” Pathway

The PPI network of the 602 DEGs^D^ revealed 5151 interactions (*p*value 1.0 × 10^−16^), with each protein in the network exhibiting an average of 17 interactions (Figure S2). The transcription factor encoded by *TP53* had the largest number of interactions (103) (Table S1). The proteins of 95 genes exhibited more than 17 interactions and considered hubs (Figure S2, Table S1). Among them, 65 corresponded to members of the superpathway (Table S1).

**Figure 5:**
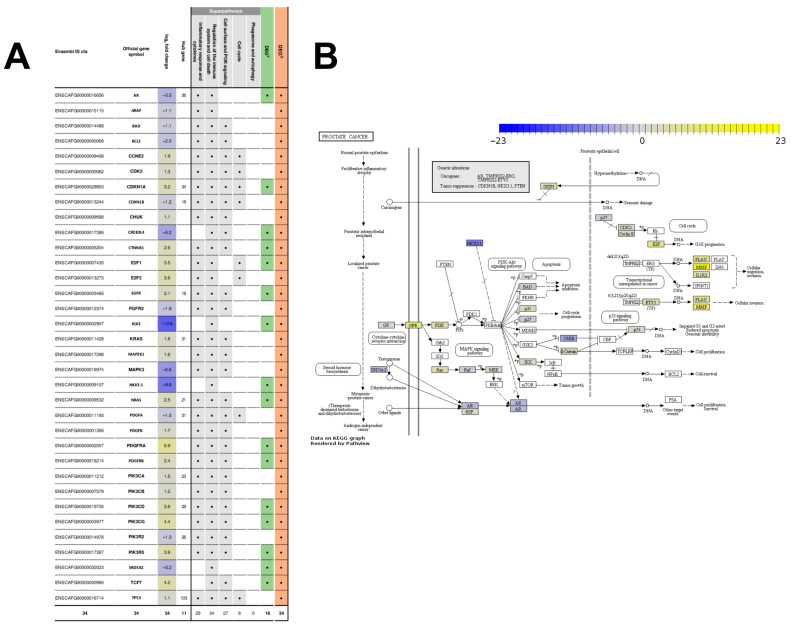
Prostate cancer (PCa) pathway. (**A**) Differentially expressed genes (DEGs) in the PCa pathway. Ensembl gene identifier (Ensembl ID cfa, column 1), official gene symbol (column 2), base-2 logarithm (log2) fold-change in the expression value of the genes between the nonmalignant and malignant sample groups (column 3); in columns 5 to 11, a dot indicates whether the gene is a database-associated deregulated gene (DEG^D^, column 11) and/or a strongly deregulated superpathway gene (DEG^S^, column 10), as well as whether the gene is a member of any of the five superpathways (columns 5–9): (i) inflammatory response and cytokines; (ii) regulation of the immune system and cell death; (iii) cell surface and PI3K signaling; (iv) cell cycle; and (v) phagosome and autophagy; (**B**) Modified graphic diagram of the PCa pathway (cfa05215; [40]), visualized using the Pathview Web tool [41]. Genes are represented as rectangles and molecular interactions as arrows. The color of the rectangles illustrates the log2 fold-changes of the expression value of the genes between the nonmalignant and malignant sample groups; upregulated genes are displayed in yellow; downregulated genes in blue; genes that are not deregulated are shown in gray. Protein–protein interactions: phosphorylation (+p), dephosphorylation (−p). Additional pathways related to the PCa are highlighted as boxes with rounded corners. Related pathways that are associated with the superpathways are highlighted with green boxes with rounded corners. Discrepancies in annotations between KEGG and the database for annotation, visualization and integrated Discovery (DAVID) are highlighted with black asterisks.

Interestingly, eleven hubs were members of the “prostate cancer” pathway; these hubs interacted with almost one third (178) of the 602 DEGs^D^. Moreover, 30 of the interaction partners of the eleven hubs that were members of the “prostate cancer” pathway were, in turn, hubs. Together, these 41 genes included members from all five superpathways, emphasizing the relevance of the “prostate cancer” pathway. Indeed, of the eight pathways that are known to actively cross-talk with the “prostate cancer” pathway (Figure 5), five were tightly connected to four of our superpathways. In particular, the “cytokine-cytokine receptor interaction” pathway is represented by the “inflammatory response and cytokines” superpathway. Similarly, the “PI3K-Akt signaling” pathway is represented by the “cell surface and PI3K signaling” superpathway. Finally, the “cell cycle”, “p53 signaling pathway” and “transcriptional misregulation in cancer” pathways are represented by the “cell cycle” superpathway. As observed for all other deregulated pathways, most (21, 62%) of the DEGs in the “prostate cancer” pathway were upregulated. Nevertheless, *AR*, the gene encoding the hub with the second largest number of interactions (36) among those in the “prostate cancer” pathway, was strongly downregulated (log_2_ fold-change −3.5).

## 3. Discussion

In contrast to its human counterpart, canine PCa is usually detected at relatively late stages [5,7]. In addition, there is no comprehensive marker set available for routine diagnostics and treatment success rates are low [2,20]. These observations point both to the need for a better understanding of the molecular mechanisms underlying canine PCa and more effective therapeutic and prognostic strategies. In our study, we applied RNA-Seq to characterize the transcriptional landscape of canine PCa as well as presenting a framework to prioritize PCa biomarker candidates to address different clinical questions. Furthermore, we integrated clinical and bioinformatic data analysis to systematically characterize the genes that are differentially expressed between malignant and nonmalignant canine prostate samples. Such multidisciplinary approaches have recently been highlighted as being of particular importance in veterinary medicine [30].

### 3.1. Five Superpathways Provide a Comprehensive Insight into the Hallmarks of Canine PCa

We were able to identify five superpathways as hallmarks of canine PCa. These superpathways summarize the essential features of the 49 pathways that were found to be deregulated in canine PCa and encapsulate the complex interactions between the tumor, its environment and the immune system. In addition to providing a comprehensive, integrative and robust approach to understand PCa biology, the superpathways open up avenues for developing new diagnostic tests and therapeutic approaches.

The transcriptional changes observed in canine PCa largely involve pathways that are relevant to the immune and inflammation responses, particularly represented by the “inflammatory response and cytokines” and “regulation of the immune system and cell death” superpathways. Indeed, the cytokine and chemokine network has been reported to support tumor development by initiating tumor growth and angiogenesis [42,43]. In addition, three of the five superpathways (“inflammatory response and cytokines”, “regulation of the immune system and cell death” and “cell surface and PI3K signaling”) reflect changes in cell adhesion molecules. Generally, cell adhesion molecules are involved in cell-to-cell and cell-to-extracellular matrix (ECM) adhesion [44] and, consequently, are important for tissue architecture [45]. Consistently, cell adhesion molecules play a decisive role in tumor invasiveness and dissemination [45,46]. As such, they are of potential prognostic value. The environment that contributes to the survival and progression of the tumor is composed largely of the ECM, inflammatory and endothelial cells and fibroblasts [47]. Therefore, the three aforementioned superpathways could provide insights into the mechanisms leading to metastasis of canine PCa.

Apart from its aforementioned role in inflammation, the “regulation of the immune system and cell death” superpathway largely involves genes such as collagen, integrin and matrix metalloproteinases, which are also involved in tissue remodeling in the canine prostate, particularly as part of the ECM or associated therewith [48]. In dogs, physiological alterations of the tissue structure depend on age, hormonal influence and neutering status [49]. Moreover, molecular profiling has also been deemed helpful for classifying prostatic lesions such as proliferative inflammatory atrophies and prostatic intraepithelial neoplasia [9]. Thus, the activation state of the superpathways, including genes associated with tissue remodeling, could be used to support clinicopathological findings in canine PCa.

In addition to being linked to cell adhesion, the “cell surface and PI3K signaling” superpathway provides insights into the activation state of the PI3K-AKT signaling pathway. This pathway has been implicated in the development of human castration-resistant PCa [50,51] and is the focus of ongoing research on pathway-specific inhibitors for this condition [52]. Current research encourages targeting PI3K signaling in combination with other compounds [53]. Several therapeutic interventions are evaluated in murine xenograft in vivo models providing valuable data [54,55,56]. Nonetheless, these models are rarely able to mimic PCa biology as closely as canine PCa is able to [3,7,8]. Therefore, the “cell surface and PI3K signaling” superpathway offers the possibility of evaluating the efficiency of these combinations in canine PCa delivering superior *in vivo* data, further underlining the relevance of the dog as a biological model for human PCa.

The “cell cycle” superpathway represents another hallmark of canine PCa. Among others, it encompasses cyclines and transcription factors that control mitosis and mediate mechanisms that are central for the balance between cell growth and death [57,58]. The potential of cell cycle genes as diagnostic biomarkers for canine PCa is supported by their use in human PCa multi-gene assays [59,60].

Finally, the smallest superpathway is associated with phagosome and autophagy. Autophagy has been repeatedly associated with the castration-resistant state of human PCa and has been linked to the emergence of therapeutic resistance [61]. This is particularly relevant to canine PCa, for which therapeutic options are limited [2].

Together, the five superpathways encapsulate the complex and cross-talking gene network of canine PCa and facilitate its molecular analysis.

### 3.2. Database-Associated Strongly Deregulated Superpathway Genes Are Reliable Biomarker Candidates for the Diagnostic and Therapeutic Work-Up

High-throughput data mining has great potential for discovering novel PCa biomarkers [35]. Our multi-step RNA-Seq screening identified 428 strongly deregulated superpathway genes (DEGs^S^) and 602 database-associated deregulated genes (DEGs^D^). A total of 169 genes were common to both gene sets, i.e., database-associated strongly deregulated superpathway genes (DEGs^SD^). While the DEGs^S^ meet statistically stringent criteria, the DEGs^D^ reflect the state-of-the-art knowledge of PCa in different databases and in the literature. The DEGs^SD^ meet both sets of criteria, thus offering a portfolio of most promising biomarker candidates to design, prioritize, implement and manage research programs addressing diverse clinical questions in canine PCa.

The strongest PCa biomarker candidates are the 16 DEGs^SD^ that are members of the “prostate cancer” pathway. These genes provide a basic but comprehensive understanding of the fundamental biological processes deregulated in canine PCa. Indeed, the 16 genes encompass genes of four of the five superpathways (all except for the “phagosome and autophagy” superpathway) and are present in multiple databases. Furthermore, half of the “prostate cancer” pathway DEGs^SD^ are well described in the PCa literature (*AR*, *CDKN1A*, *CTNNB1*, *EGFR*, *KLK2*, *NKX3-1*, *PDGFRA*, *SRD5A2*), confirming that this is a pivotal pathway to specifically target clinical questions on canine PCa. Finally, the “prostate cancer” pathway also includes DEGs^SD^ that are relatively specific to the (human) prostate (*SRD5A2*, *KLK2*, *NKX3-1* and *CREB3L4*), proto-oncogenes (*EGFR*, *PDGFRA*, *PDGFRB*, *NRAS*) and druggable candidates (*PIK3CD*, *CTNNB1*, *PIK3CG*, *CDKN1A*). Notably, the “prostate cancer” pathway comprises *KLK2*, the most strongly downregulated DEG^SD^ (log_2_ fold-change −12.6). *KLK2* is of particular interest because it has a one-to-many orthologous relationship to human *KLK3*, the gene encoding the PSA [23]. Nevertheless, it is worth noticing that, in contrast to human PSA, *KLK2* was down-regulated in our study and that the serum levels of CPSE, the protein encoded by *KLK2*, have been reported to exhibit the same trend [25]. Beyond the use of the kallikrein members as diagnostic markers for human PCa [62], the kallikrein network is involved in a variety of processes potentially driving tumor progression [63]. Hence, the down-regulation of *KLK2* could still be of diagnostic or therapeutic significance. Supporting the role of kallikrein members in canine PCa, we also identified *KLK4*, another member of the kallikrein family, as down-regulated. The role of *KLK2* and *KLK4* in canine PCa certainly warrants further investigation.

Thirteen DEGs^SD^ are the canine orthologs of genes used in human commercial PCa multi-gene assays. This is of peculiar importance because there is a possibility to rapidly adapt molecular screening assays used in humans for use in dogs. Strikingly, these 13 DEGs^SD^ encompass genes of all five superpathways and, hence, represent the disease hallmarks of canine PCa in a comprehensive manner. Moreover, the 13 DEGs^SD^ could be used to systematically evaluate the dog as a model for human PCa.

Two additional groups of genes that could complement the aforementioned ones as diagnostic biomarkers for canine PCa are prostate-specific genes and oncogenes. Overall, we were able to assign nine DEGs^SD^ as being relatively specific to the (human) prostate, and hence, as potential specific markers for the canine prostate. In the diagnosis of human PCa, specific markers are utilized in immunohistochemistry to confirm the prostate origin of the sample [64,65]. On the other hand, oncogenes are known to be pivotal for tumorigenesis [47]. In human castration-resistant PCa, oncogenes have been suggested to control a variety of pathways [66] and shown to activate the tyrosine kinase network [67].

Furthermore, we identified several DEGs^SD^ that bear the potential to improve and scale up the use of chemotherapeutic agents and molecularly targeted therapeutic strategies for canine PCa. Notably, 17 upregulated DEGs^SD^ encode tyrosine protein kinases. Tyrosine kinases have become the subject of growing attention in veterinary medicine regarding molecular targeted therapies [68]. In particular, the two tyrosine kinase inhibitors (TKIs), toceranib and masitinib, have been approved as molecular targeted therapies for canine patients [68]. Among other receptor tyrosine kinases, toceranib and masitinib both target the tyrosine kinase KIT [69,70,71], which has been frequently described as a prognostic marker and therapy target, especially in canine mast cell tumor [72]. *KIT* was a strongly upregulated DEG^SD^ (log_2_ fold-change 4.1). Previous findings on the canine prostate indicate that samples with a high Gleason score are positive for immunoexpression of c-kit [11]. Therefore, such cases could benefit from the use of a TKI. Whether the dog can provide additional therapeutic insights for human PCa still needs to be evaluated.

In addition to the aforementioned therapeutic potential, some DEGs^SD^ have prognostic value. For example, high levels of the multi-resistant p-glycoprotein *ABCB1* (also known as *MDR1*) have been associated with ABC-protein-mediated chemotherapeutic resistance of tumor cells [73] and may explain the limited efficacy of doxorubicin on canine PCa cell lines [74]. Current therapeutic options for canine PCa are limited [2]. The data presented here provide an illustration of how our RNA-Seq data may contribute to the development of new therapeutic strategies to improve treatment outcome.

### 3.3. Database-Associated Deregulated Genes Such as TP53, MYC and AR Are Crucial to Complement the DEGs^SD^ and Tackle Clinically Relevant Questions

To date, 433 DEGs are present in public databases [40,75,76,77] or have been reported in the literature [59,60,78] and are therefore DEGs^D^ but did not satisfy the criteria of the DEGs^S^. Some of these genes have been extensively evaluated and characterized in both canine and human PCa. The most noteworthy among them is perhaps *TP53*. *TP53* is a druggable member of the “prostate cancer” pathway that has been frequently reported in the literature as pivotal in the tumorigenesis of canine PCa [79]. Consistent with this hypothesis, we found that *TP53* has the largest number of interactions within the deregulated gene network of canine PCa. However, it was only moderately deregulated (log_2_ fold-change 1.1) between malignant and nonmalignant canine prostate samples. Similarly, *MYC* is a druggable proto-oncogene frequently discussed in the literature [80] but was only moderately deregulated (log_2_ fold-change 1.5). Such examples demonstrate the importance of the DEGs^D^ as complement of the DEGs^SD^ to customize and target different aspects of canine PCa.

Ultimately, a panel combining DEGs^SD^ and DEGs^D^ appears as the most appealing option to tackle unanswered questions on the hormonal axis in canine PCa. In dogs as in humans, steroid hormones such as androgens and estrogens generally affect the prostate gland development and functionality [7,81,82], which is why their deregulation is expected to have a major impact. Indeed, almost all human PCas are acknowledged to begin in an androgen-dependent state in which androgen deprivation therapies are standard and have proven effective [83]. In contrast, androgen deprivation therapies are not successful in dogs [84]. In this context, we found that 16 DEGs^SD^ (*BRCA1*, *CASP8*, *CAV1*, *CDC6*, *CDK1*, *CTNNB1*, *E2F1*, *EGFR*, *ETV1*, *KLK2*, *KPNA2*, *NKX3-1*, *PROM1*, *RUNX2*, *SLC45A3*, *TMPRSS2*) and 20 DEGs^D^ (*AREG*, *CAPZA1*, *EZH2*, *FOLH1*, *FOXA1*, *GEN1*, *HSP90B1*, *IL6*, *KLF4*, *KLK4*, *KRAS*, *KRT5*, *MAPK14*, *MKI67*, *MYC*, *NCOA1*, *NR3C1*, *TNF*, *TNK2*, *UBE2C*) are direct interaction partners of the *AR*. Although the majority of the direct interaction partners of the *AR* were upregulated, the *AR* itself was downregulated. This might be due to differences in the number of castrated dogs between the malignant (7/10) and the nonmalignant (0/14) sample groups. Nevertheless, our observations are supported by a recent study suggesting that canine PCa is associated with loss of AR expression [82,85]. In addition, we found that the estrogen receptor 2 (*ESR2)* and the steroid 5 alpha-reductases 1 and 2 (*SRD5A1* and *SRD5A2)* were downregulated. *SRD5A1* and *SRD5A2* encode the 5α-reductases, which are involved in the transformation of testosterone to 5α-dihydrotestosterone [86] and, like estrogens, actively regulate the size of the prostate [81,82]. DEGs^SD^ such as *NKX3-1* and *KLK2* and also DEGs^D^ such as *ACPP* (also known as *PAP*) and *FOLH1* are among other genes known to be regulated by the AR and, thus, presumably relevant to the hormonal axis of canine PCa. Therefore, we found that the tumor suppressor *NKX3-1* was strongly downregulated in the malignant sample group (log_2_ fold-change −9.8). This result is in agreement with previous research on progressive canine PCa [80]. Moreover, *NKX3-1* has been recommended as a marker to determine prostatic origin of metastatic tumors [87]. We also observed that *ACPP* was strongly downregulated (log_2_ fold-change −5.1). *ACPP* encodes a prostate-specific acid phosphatase that is orthologous to a human prostate-specific tumor suppressor [88] reported to be affected by the exposure to androgens in human PCa [89]. Consistent with a tumor suppressor role for *ACPP* in the canine prostate, positive immunostaining of prostate-specific acid phosphatase has been associated with normal prostatic acinar tissues in dogs [90]. In contrast, we observed that *FOLH1* was strongly upregulated (log_2_ fold-change 4.0) in the malignant sample group. *FOLH1* encodes the prostate-specific membrane antigen (PSMA) that is orthologous to an androgen-repressed gene in human PCa [91]. This is in agreement with our findings and research from others, who detected that *FOLH1* is expressed in AR-negative canine PCa cells [92]. Overall, these examples demonstrate the complex gene interactions that need to be understood to answer clinical questions and further compare human and canine PCa.

## 4. Materials and Methods

### 4.1. Ethical Statement and Sampling of Fresh Frozen Prostate Tissue and FNA Samples

In the present study, a subset of samples was analyzed (Figure 1), which was previously used to evaluate similarities and differences at gene expression level, comparing FNA samples with fresh frozen canine prostate tissues [37]. Samples used in the present study were collected in agreement with the owners. None of the dogs were euthanized due to reasons of sample collection. Aspirates of the canine prostate collected by ultrasound-guided FNA were obtained at the Small Animal Clinic of the University of Veterinary Medicine Hannover, Foundation (Germany) in accordance with the German Animal Welfare Guidelines, approved by the Ethics Committee of the State of Lower Saxony, Germany (No. 14/1700).

### 4.2. Data Processing and Differential Gene Expression Analysis

Raw sequencing reads were obtained from the Gene Expression Omnibus database (GSE122916) [37,93]. One sample (US-3) was excluded from the analysis. The malignant sample group comprised nine fresh frozen prostate tissues and one aspirate collected using the ultrasound-guided FNA technique. Nonmalignant samples of the canine prostate were used as control, including nine fresh frozen tissue samples and five FNA samples. The diagnosis of the fresh frozen tissue samples was based on histopathological examination, while the samples collected by FNA were examined cytologically (Figure 1) [37]. Quality control of raw sequencing reads were initially performed with FastQC [94]. Raw reads were mapped to the dog CanFam3.1 reference genome assembly and annotation of the Ensembl database (version 94, [95]) using STAR (version 2.5.0) [96] with parameters “--sjdbGTFfile Canis_familiaris.CanFam3.1.94.gtf --sjdbOverhang 70 --quantMode TranscriptomeSAM GeneCounts --quantTranscriptomeBan IndelSoftclipSingleend”, where “Canis_familiaris.CanFam3.1.94.gtf” is the genome annotation downloaded from the Ensembl database (http://ftp.ensembl.org/pub/release-94/gtf/canis_familiaris/Canis_familiaris.CanFam3.1.94.gtf.gz, last accessed on 18 October 2021). Count data for Ensembl identifiers were normalized for multiple testing with the R/Bioconductor package DESeq2 [97], and differentially expressed genes (DEGs) were identified. Genes with a false discovery rate (FDR) ≤ 1 × 10^−5^ were considered to be differentially expressed. Ensembl Compara [98] was used to identify *homo sapiens* (hsa) orthologs to *canis lupus familiaris* (cfa) genes, and thus improve the limited annotation of the dog genome [30].

### 4.3. Pathway Enrichment and Functional Analysis

Pathway enrichment and functional analysis of DEGs were performed with DAVID (Database for annotation, visualization and integrated discovery tool) [38] based on the KEGG_PATHWAY (Kyoto Encyclopedia of Genes and Genomes) [40] and GOTERM_BP_FAT (Gene Ontology Biological Processes). The cut-off for statistical significance was an FDR of less than or equal to 5%. The analyses were performed for the human orthologs of the canine genes, which were retrieved using Ensembl Biomart (version 94) [99]. The background for the analysis was the set of 19,324 human genes that were reported as orthologs of one or more canine genes by Ensembl Biomart. Enriched GOTERM_BP_FAT terms were summarized with REVIGO (Reduce + Visualize Gene Ontology) [39].

### 4.4. Pathway Cross-Talk

Cross-talk between the KEGG pathways that were enriched among the differentially expressed genes was quantified based on their gene overlap. Specifically, we computed the overlap coefficient between all pairs of pathways. Then, we hierarchically clustered the pathways using the complete linkage algorithm based on a distance defined as 1 minus the overlap coefficient. KEGG pathway annotation for each gene was retrieved from DAVID [38].

### 4.5. Multi-Step Screening for Canine PCa Biomarker Candidates

A multi-step screening approach was used for identification of “strongly deregulated superpathway genes” (DEGs^S^) and “database-associated deregulated genes” (DEGs^D^). A gene was a DEG^S^ (Figure 1) if (i) its base-2 logarithm (log_2_) fold-change was smaller than −2 or larger than 2; (ii) its normalized count was above the 40th percentile in at least one of the sample groups; and (iii) it was a member of the *superpathway* (i.e., a member of one of its constituent KEGG pathways).

A gene was a DEG^D^ (Figure 1) if (i) it was enriched (11), group enriched (25), and/or elevated (84) in the prostate according the human protein atlas (https://www.proteinatlas.org/humanproteome/tissue/prostate, last accessed on 18 October 2021 [75]); (ii) it was a member of the KEGG “prostate cancer” pathway (cfa05215, [40]); (iii) it was a proto-oncogene (keyword:”Proto-oncogene [KW-0656]” AND organism:”Homo sapiens (Human) [9606]”) or a tyrosine-protein kinase (keyword:”Tyrosine-protein kinase [KW-0829]” AND reviewed:yes AND organism:”Homo sapiens (Human) [9606]”) in the UniProt keyword database (https://www.uniprot.org/, last accessed on 18 October 2021 [76]); (iv) it was reported by PubMed (https://www.ncbi.nlm.nih.gov/pubmed/, last accessed on 18 October 2021) literature mining ((dogs[MeSH Terms]) AND prostatic neoplasms[MeSH Terms] AND biomarkers[MeSH Terms]) OR (canine[TIAB] OR dog[TIAB] AND prostate cancer[TIAB]) between 2015–2020 and additional manual curation of “canine prostate”; (v) the tissue-based human PCa multi-gene assays Oncotype, Prolaris, and Decipher [36,59,60,78]; and (vi) analyzed on drug-gene interactions using the Drug-Gene Interaction Database with official gene names and the filter “FDA approved”, “antineoplastics” and “immunotherapies” [77]. DEGs that were assigned to at least one of the aforementioned criteria were designated as database-associated DEGs^D^. DEGs^D^ that additionally matched the criteria as DEGs^S^ were termed as “database-associated strongly deregulated superpathway genes” (DEGs^SD^).

### 4.6. PPI Network Construction and Identification of Hub Genes

The Search Tool for the Retrieval of Interacting Genes/Proteins (STRING) database, version 11.0 [100], was employed to construct a protein–protein interaction network (PPI) for the 602 genes in either DEG^S^ and/or DEG^D^. PPIs with a confidence greater than 0.4 were considered reliable. DEGs of the “prostate cancer” pathway and associated level of deregulation were visualized using the Pathview Web tool [41].

## 5. Conclusions

In conclusion, to the best of our knowledge, this is the first tissue- and biopsy-based study comparatively characterizing the transcriptional landscape of canine PCa samples and samples of nonmalignant origin. In addition to identifying five superpathways encapsulating the hallmarks of canine PCa, we provide a framework for prioritizing candidate canine PCa biomarkers for different purposes. It is noteworthy that *KLK2* featured recurring significantly in our assessment: it is the most strongly downregulated DEG^SD^, a member of the “prostate cancer” pathway, a putative prostate-specific marker and it is involved in the hormonal axis of PCa. Although further studies are necessary to confirm *KLK2* as a clinical marker, *KLK2* illustrates the importance of integrating clinical information such as medical history, age, hormonal status and cytological or histopathological diagnosis with molecular biological information. In summary, our data are a valuable resource for the diagnostic, prognostic, and/or therapeutic work-up of canine PCa, and an orientation for further gene expression studies, such as targeted NGS or qPCR screenings.

## Data Availability

The dataset analyzed in this study is openly available in the Gene Expression Omnibus database (https://www.ncbi.nlm.nih.gov/geo/, last accessed on 18 October 2021), accession GSE122916.

## Supplementary Materials

The following are available online at https://www.mdpi.com/article/10.3390/ijms222111481/s1.
