# Supplementary material for: An RNA-Seq-Based Framework for Characterizing Canine Prostate Cancer and Prioritizing Clinically Relevant Biomarker Candidate Genes
Source: Int J Mol Sci. 2021 Oct 25;22(21):11481. doi: 10.3390/ijms222111481 (PMC8584104; doi:10.3390/ijms222111481)
Supplement: Supplementary file 1 [file ijms-22-11481-s001.zip › Supplementary files/Figure S1.pdf]

A

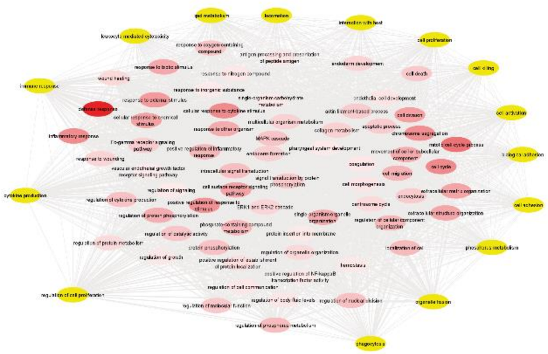

B

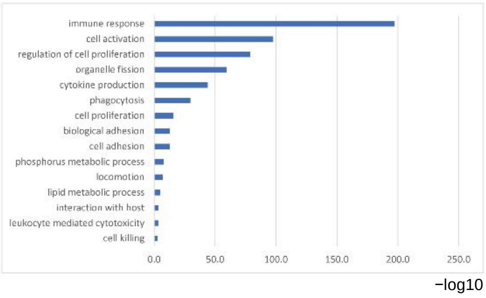

Figure S1: Enrichment analysis of Gene Ontology (GO) biological processes (GOTERM_BP_FAT) among canine PCa DEGs summarized and visualized using REVIGO. (**A**) Bubble plot of the GOs. The color and size of circles indicate the log10 of the false discovery rate (FDR) and the frequency of the GO term, respectively. Representative terms are highlighted with yellow oval circle (**B**) Bar chart summarizing the biological processes. The height of the bars denotes the sum of the -log10 FDR of the individual biological processes clustered together by REVIGO and represented by the respective terms on the x-axis.
