# Supplementary material for: An RNA-Seq-Based Framework for Characterizing Canine Prostate Cancer and Prioritizing Clinically Relevant Biomarker Candidate Genes
Source: Int J Mol Sci. 2021 Oct 25;22(21):11481. doi: 10.3390/ijms222111481 (PMC8584104; doi:10.3390/ijms222111481)
Supplement: Supplementary file 1 [file ijms-22-11481-s001.zip › Supplementary files/Figure S2.pdf]

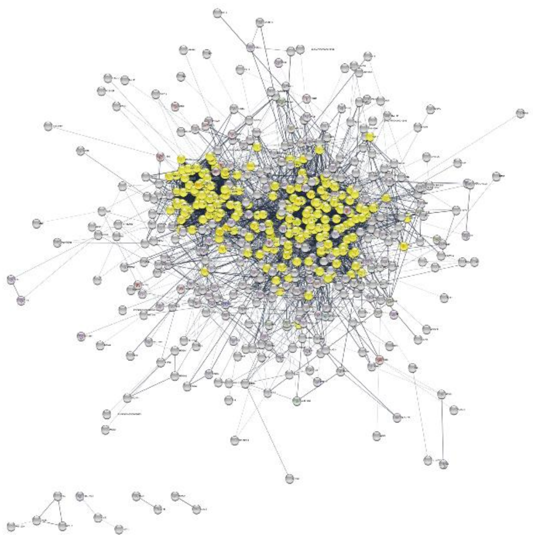

Figure S2: The protein interaction network of the 602 database-associated deregulated genes (DEGsD). The DEGsD are displayed as nodes in a network, where connections between nodes represent protein–protein interactions or functional relationships, according to the STRING database [101]. Genes with more than 17 interactions were defined as hub genes and are indicated in yellow.
